# Real-Time Scheduling in IoT Applications: A Systematic Review

**DOI:** 10.3390/s23010232

**Published:** 2022-12-26

**Authors:** Sima Abolhassani Khajeh, Morteza Saberikamarposhti, Amir Masoud Rahmani

**Affiliations:** 1 Department of Computer Science, Islamic Azad University, Qeshm International Branch, Qeshm 79515/1393, Iran; 2 Department of Computer Engineering, Islamic Azad University, South Tehran Branch, Tehran 1584743311, Iran; 3 Future Technology Research Center, National Yunlin University of Science and Technology, Douliu 64002, Taiwan

**Keywords:** Internet of things (IoT), scheduling, real-time, fog computing, cloud computing

## Abstract

The Internet of Things (IoT) is a telecommunication network in the next generation of applications with the rapid progress of wireless sensor network techniques that have touched many spheres of life today. Hardware, telephony, communications, storage, secure platforms, software and services, and data processing platforms are all part of the IoT environment. IoT sensors collect data from their environment and share it by connecting to the Internet gateway. These sensors often perform tasks without human intervention. This article aims to review real-time scheduling in the IoT to fully understand the issues raised in this area published from 2018 to 2022. A classification for IoT applications based on practical application is provided for selected studies. Selected studies include healthcare, infrastructure, industrial applications, smart city, commercial applications, environmental protection, and general IoT applications. Studies are sorted into groups based on related applications and compared based on indicators such as performance time, energy consumption, makespan, and assessment environments depending on the provided classification. Finally, this paper discusses all reviewed studies’ main concepts, disadvantages, advantages, and future work.

## 1. Introduction

The IoT is a system of integrated computations between digital and mechanical sensors that can provide data transmission through networks without human interposition [1]. Things in the mentioned system can be remotely controlled by the infrastructures of networks [2]. IoT is currently in a diverse range of applicable plans for education, healthcare, agriculture, the army, and industries [3]. Due to the large amount of information generated daily from IoT devices, real-time scheduling and fast data processing are essential [4]. Industrial plans often need fault-tolerant guarantees and reliable practices while simultaneously having immediate performances. In general, it is not possible to fulfill all these requirements simultaneously. A practical task can be divided into several tasks requiring a particular computation capability. There are two duty-allocation sections, i.e., allocating duties to processing units and scheduling tasks, i.e., task prioritizing work performance and data transmission arrangement between the processing units [5].

Scheduling is an approach through which duty selection takes place on a priority basis and selects the best option to perform the task [6]. Efficient scheduling can lead to a swift on-time response favorable in intelligent systems. For instance, a prompt notification system is required to save a patient in a smart health care system. Efficient scheduling algorithm development is necessary to maximize the use of devices with resource limitations. The goal is to reduce reaction time and take advantage of the network with no energy consumption enhancement [7]. This paper attempts to study the immediate scheduling of the IoT. Immediately scheduling an event commissioning that decides which controlling statement must be performed at each moment is constructed by periodic assessing of the common distribution of the momental phase normalized weight functions sampled at every moment [8].

Developments occur in terms of technology and embedded systems of the IoT, and considering time limitations or certain deadlines, responding in real-time and immediate computations are of significant importance. Therefore, task scheduling is vital to determine the best arrangement to perform the duties and use the existing resources. Immediate systems scheduling is divided into immediate hard and soft systems scheduling. If this system’s tasks have a period and are completed after the deadline, the task is not applicable. A soft real-time system is one in which the tasks are flexible and have fewer deadlines. Aviation systems, nuclear power reactors, and anti-lock brake systems are examples of hard instantaneous systems for autos. The options are soft real-time systems, multimedia playback systems, and automatic windshield wipers [6]. The article [6] proposed a bespoke adaptive and smart scheduling algorithm for optimal performance and management of the soft and hard real-time activities implemented in the IoT systems. In the article [9], in the form of a processing unit, a creative job scheduling was presented that is ideal for real-time embedded systems. Research is focused on a scalable approach definition based on extending the existing Earliest Deadline First (EDF)-based scheduling to the Guaranteed Earliest Deadline (GED) algorithm-based scheduling performed in four phases.

From the standpoint of chip costs, the proposed scheduling is scalable to increase a system’s maximum number of activities. In the article [10], the problem of immediate traffic scheduling with difficult deadlines in a temporary wireless network was reviewed and studied. The article aims to present guarantees regarding the on-time output and freshness of data from the sight of the Age of Information (AoI), a new criterion. In this task, the real-time traffic algorithm was suggested for the immediate traffic plan with difficult deadlines in temporary networks, which offers guarantees regarding the on-time operative capability and freshness of data from the sight of AoI. The focus of this research is to look into real-time IoT scheduling to fully comprehend the challenges raised, such as scheduling and service algorithms and the primary IoT applications in this field. In addition, we look at open issues and future work. First, we describe the evaluation factors used in the article in Table 1. The main contributions of this paper can be listed as follows:

- Creating a classification system for real-world IoT applications;
- Discussing the challenges of task scheduling in the IoT.
- Expressing future projects and issues around IoT real-time scheduling that is mandatory to be addressed.

**Figure 1:**
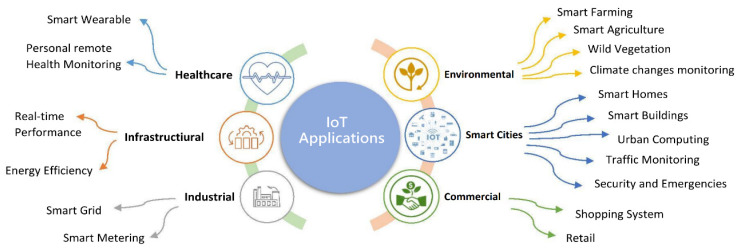
The IoT applications.

Figure 1 depicts IoT applications:

As shown in Figure 1, IoT is used in various fields. Among the IoT applications in health care that can be mentioned are health, management, and clinical services supervision utilizing patients’ physiological information through sensor nodes that connect the patient to various controlling devices [19]. In [20], an IoT architecture is offered for the early detection of patients who suffer from cardiovascular diseases through a machine learning algorithm. This architecture includes three layers to collect the sensor data from wearing devices, store data in the cloud, and create a predicting model based on the regression for cardiovascular patients. In real-time, the IoT can be used in environmental programs to produce dense maps of air, water, and sound pollution. There are different IoT applications in a smart city. For example, it includes home automation, health care via cellphones, production automation, assisting the elderly, medical aids, vehicles, intelligent networks, smart energy control, traffic management, etc. [21]. The IoT can also be used to supervise and enhance the awareness of building residents about energy consumption and automate the lighting systems of commercial facilities and factories [22]. The Industrial IoT (IIoT) goal is goods production, smart factories, and making connections between customers and commercial partners. The IoT has been the area of attention in academic and industrial areas; however, the risk of privacy protection and poor security is due to the lack of substantial security technology [23]. To minimize the job application delays and increase the operational capability in terms of response time, in health care tasks, and to reduce delays, it is required to process jobs using immediate timing methods and maximize the medical operational capability [11]. Immediate timing can be utilized in the imaging processes of optical satellites. Conventional offline timing causes wastage of computational resources, and due to the dynamic environmental changes, immediate timing is needed to optimize and decrease computational costs [24]. In smart buildings, an efficient schedule is used to save energy consumption and user convenience and reduce energy costs [25]. In traffic lights in a smart city, using the technique of real-time scheduling and adjustment of traffic signals according to the input information, the calculations of the generated data are performed immediately. As a result, traffic load, pedestrian congestion, and traffic costs are reduced [26].

## 2. Related Work

Because real-time systems react to real-world events over a while, they are different from non-real-time systems. These systems aim to observe and control external processes and respond on time, and respond to changes, sometimes in milliseconds. The fast-enough response is important for simple enough processes, but in addition to the need for complex coordination for many more complex real-time systems, rapid response to events is also challenging. This provides a study of methods for scheduling different real-time events [1] for processing important time events, and the availability of responses for real-time systems. Failure to respond promptly to these events can have serious consequences, such as system failure and even loss of life and property.

The deadline’s nature and arrival characteristics are two important features of a task. Three modes can be considered to achieve the task: a period with a fixed time interval, static distribution, and the random nature of the time between arrival. A task with a time limit is important, and must be completed before the deadline. The nature of the deadline depends on the real-time system. In tasks with a hard deadline, completing it before the specified time is so critical that if the deadline is missed, the task is no longer relevant. The loss of a hard deadline always leads to system failure. Moreover, tasks that can be performed gradually over a period of time are called soft deadlines [2]. Scheduling constraints on such tasks are very flexible, and missed deadlines do not result in irreparable failure [3].

In [3], a system for home automation consisting of microcontrollers and smartphones is described. Home equipment is controlled and observed through various communication techniques by smartphone applications. This article compares various communication techniques, including ZigBee, Wi-Fi, Bluetooth, EnOcean, and GSM. This security system is designed so authorized users can only use the system. The researchers also surveyed to find the topography of the equipment employed in the automation system in a home. In addition, a comparison of the suggested programming languages for several systems based on ECA structures and their diversities was performed.

Furthermore, the application of a pulse width modulation technique and temperature sensor to measure room temperature and change the fan speed according to temperature was suggested in a new method for communication between a temperature sensor and a microcontroller [4]. Despite the specific limitations, it has important advantages such as simplicity, cost-effectiveness, and automatic control.

Scheduling algorithms, one of the most important issues of real-time systems, can be set in single-processor scheduling, centralized multi-processor scheduling, and distributed scheduling, depending on the system environment [5]. Among the algorithms discussed in real-time scheduling are RMS, EDF, and LLF for single-processor systems, thinking and strategy scheduling for multiprocessor systems, and GRMS and DSr for distributed real-time scheduling algorithms. Minimum response time, maximum throughput, minimum overhead (memory, disk, and CPU usage), and maximum fairness are four important features of an ideal scheduling algorithm [6].

Task scheduling is one of the most important challenges in cloud computing, as service providers must serve the applications of many users at different times and from different locations flexibly and quickly. Many metaheuristic task scheduling algorithms, which are based on PSO and GA, have been suggested [8,9,10]. In [19], a task scheduling algorithm is presented by combining the cuckoo algorithm with the PSO algorithm to introduce an optimal task schedule in the cloud computing environment to complete the performance of tasks in the shortest possible time and to satisfy resource utilization. A task scheduling algorithm based on the PSO algorithm is recommended in [5]. This algorithm’s main principle is sorting the existing tasks and processors in ascending order. Existing processors are sorted by their processing power. Then, the assignment of tasks is performed using one-to-one mapping, and the Fastest Work Processor (SJFP) algorithm is employed to produce the population for the PSO algorithm initially.

In [20], an advanced work scheduling algorithm is introduced by integrating the PSO algorithm with the annealing algorithm simulated with its fast randomized, global search scheduling strategy in the cloud computing environment. Experiment results show a decrease in the average execution time and an increase in the resource availability rate. In [21], a work schedule was proposed using Hassan. Elitism is selecting better people with a tendency towards better people. Elitism is important for providing better solutions over time, speeding up PSO performance, and preventing the best solution’s loss.

## 3. Research Selection Method

IoT scheduling issues are investigated in this study using the SLR method as a research method [27].

By asking the following analytical questions and answering them, the objectives of this study are achieved:

- AQ1: What are the practical areas of real-time scheduling in IoT?
- AQ2: How are real-time schedulings in the IoT evaluated?
- AQ3: What are the most prevalent criteria for IoT real-time scheduling evaluation?
- AQ4: What are the future directions and open perspectives for IoT applications?

**Figure 2:**
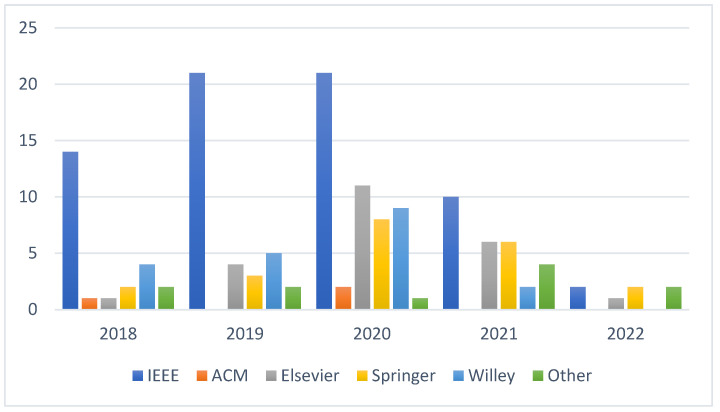
Classifying published articles according to their year and publishers.

Figure 2 depicts an IoT of studies undertaken by various academics and published in multiple databases such as Elsevier, IEEE, Springer, ACM, Wiley, and MDPI by year.

The inclusion/exclusion criteria for the final research selection were used when the analytical questions were provided. Regarding the number of papers published, we only look at journal publications indexed in WoS and ISI proceedings as peer-reviewed papers. Finally, 35 peer-reviewed studies were chosen to examine and respond to the AQs listed above, detailed in Section 4.

**Figure 3:**
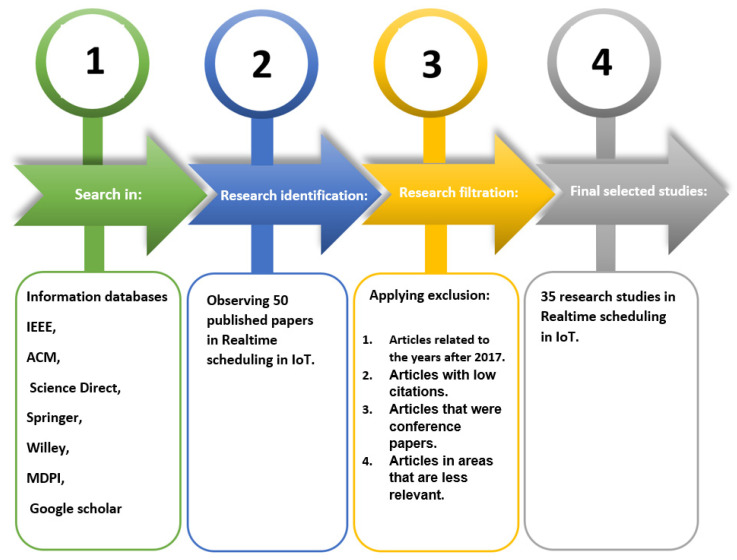
Study selection of the real-time scheduling in IoT.

Figure 3 shows how to select the number of articles studied.

**Figure 4:**
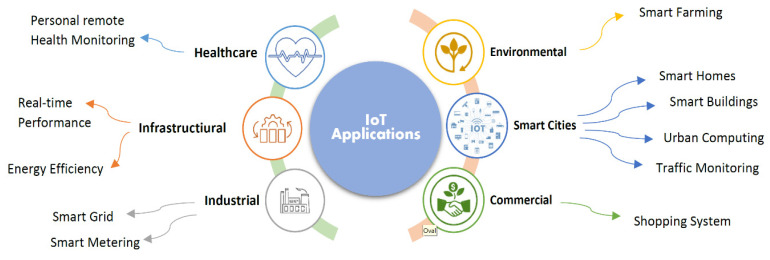
The classification of IoT applications.

Figure 4 shows the classification of IoT applications based on the reviewed articles:

The following principles were used to create the final study pattern:

- Selected articles between 2018 and 2022;
- Real-time scheduling articles on IoT topics;
- High-quality technical articles.

The following elimination process is used to acquire selected studies:

- Articles related to the years after 2017;
- Articles with low citations;
- Articles that were conference papers;
- Articles in areas that are less relevant.

## 4. The IoT Applications Organization

### 4.1. Urban Computing Applications

Two scheduling algorithms based on an Integer Linear Programming (ILP) formula, including an optimal algorithm, CASSIA-INT, and an approximate algorithm, CASSIA-RR, are proposed in [17] to lessen the makespan of fog computing applications. Mentioned schedulers look for a schedule with the tiny possible makespan while the CASSIA-RR accelerates it. These scheduling algorithms have shown different efficiencies in several scenarios, with several numbers of processing nodes and different usage levels. Compared with common algorithms for scheduling, such as Random and Round Robin, the CASSIA-INT and CASSIA-RR algorithms show better results in makespan, not violating quality of service (QoS). Because the CASSIA-INT and CASSIA-RR algorithms differ from the previously proposed methods in that they consider the CoS of the application for deciding where a program’s tasks can process, they can easily be used in resource management in a cloud–fog system. It can be inferred from the results that for all scenarios, the makespan values gained from these two scheduling algorithms were smaller than a serial execution. Moreover, a vital feature of the CASSIA-RR method is that the time required to generate a schedule in this method is significantly less than the timing required by CASSIA-INT, which is a great feature for real-time environment schedulers.

In this paper, the one-to-one execution of the application’s tasks is considered an assumption. The next assumption is to enable the virtual machines (VMs) in which tasks are to be executed before a task reaches the processing host. Fog and cloud resources’ availability, contain elements of processing and links of a network. The CoS is an application that is allocated using a classifier, and a DAG characterizes the workflow that includes the processes to be performed, the RAM, storage, and communication needs of the tasks that the application contains, as the schedulers receive input data.

While none of the prior proposed methods for task scheduling in cloud–fog systems have considered CoS of applications for deciding on the position of tasks processing, in this research two algorithms for scheduling are based on the integer linear programming formula. These two algorithms solve the exact integer programming formula and a relaxed version of the integer programming formula, respectively. Given the importance of quick response in real-time systems, the use of the CASSIA-RR algorithm, which produces approximate results close to the results provided by CASSIA-INT in a much smaller period, is highly suggested [17].

The study by [28] proposed a real-time heterogeneous hierarchical scheduling (RTH^2^S) algorithm for real-time tasks in a heterogeneous integrated fog–cloud architecture. In the study, a hierarchical model for fog nodes was considered so that nodes in higher tiers have more computing capacity and naturally have more latency from data production sources than nodes in lower tiers. In this research, tasks with different profiles are considered. The least laxity first (LLF) algorithm finds the best fog node for scheduling in regular profile tasks. In contrast, the tasks are split, or the LLF heuristic was used based on their tag values in profiles with a tag to complete execution while the deadline was not reached. The real-time efficiency of RTH2S was evaluated and validated compared with other algorithms.

The main points discussed in the paper are as follows:

1. Considering sensor heterogeneity, a multi-tier hierarchical real-time scheduling algorithm (RTH^2^S) was proposed with mathematical modeling of an n-tier fog–cloud architecture that schedules the tasks to fog–cloud processors and satisfies their requirements of the deadline;
2. Regular and tagged task profiles are both considered in the proposed algorithm;
3. Also, finding the best fog node for executing or splitting the task due to a combination of its deadline requirements and size was employed.

#### Discussion of Urban Computing Applications

Table 2 summarizes the articles’ classification mentioned above and the key criteria to consider when evaluating the urban computing strategy in IoT applications. In this method, the most crucial context is providing a scheduling algorithm to minimize cloud computing applications’ length and an algorithm in a multi-layer fog network with different job profiles that schedule real-time tasks.

Table 3 outlines a method for evaluating the above research utilizing the assessment factors in IoT urban computing applications. The parameters are effectiveness, efficiency, makespan, execution time, success ratio, task load, propagation delay, heterogeneity level, deadline factor, and monetary cost. Most research studies in the urban computing method evaluated their offered approach in the effectiveness, execution time, makespan, cost, and latency characteristics.

### 4.2. Traffic Monitoring Applications

A strategy for scheduling traffic signals and a macroscopic model for a mixed flow network for pedestrians and vehicles were presented in [29]. A new mathematical model of pedestrians crossing an intersection was first proposed. Then, to provide the right balance between the needs of pedestrians and the needs of vehicle drivers, and with a combination of a link-based vehicle network model, a traffic light scheduling problem was formulated. The method of solving this article for this problem was performed in two phases:

1. First, the problem was transformed into a complex mixed linear programming (MILP) problem through a new conversion method that can be solved using an existing solver, such as GUROBI;
2. In the next part, to reduce the computational complexity in MILP, a meta-heuristic method called Discrete Coordination Search Algorithm (DHS) was developed.

The most important accomplishments of this paper are the presentation of two new methods to solve the traffic signal scheduling problem called the Mathematical-Based Algorithm (MILP) and the Evolutionary Algorithm (DHS). The experimental results presented show a considerable increase in the efficiency of the DHS algorithm while it can guarantee an accepted level of proficiency degradation. Furthermore, a comparison between our strategy and the mixed-stream optimization strategies with the wide isolated intersection in the commercial Vissim simulator shows the better performance of the method in this paper compared with other available methods. Finally, a quantitative analysis of the effect of pedestrians on the vehicle traffic network was presented by presenting various important levels on the pedestrian side.

#### Analyzed Traffic Monitoring Applications

Table 4 summarizes the papers’ classification mentioned above and the key criteria to consider when evaluating the traffic monitoring strategy in IoT applications. This method’s case studies include the following: (1) analysis of a simplified traffic network profile using weights; (2) a bidirectional four-way pedestrian-vehicle mixed-flow network.

Table 5 outlines a method for evaluating the above research utilizing the assessment factors in IoT traffic monitoring applications. The analysis considers the following parameters: traffic costs, harmony memory size, efficiency, harmony memory considering rate, and pitch adjustment rate. Most research studies on the traffic monitoring method evaluated their offered approach in the cost characteristics.

### 4.3. Smart Grid Applications

In the paper by [14], the Time Slotted Channel Hopping (TSCH) method, which is one of the new medium access control (MAC) protocols, was used to improve network scheduling to minimize latency and maximize throughput. Moreover, the Neurogenetic Algorithm (NGA) was used for TSCH scheduling through focus node scheduling to utilize the channel properly. Performance evaluation of this method in real-time IoT sensors with time latency and throughput parameters was performed and compared with handed TSCH scheduling algorithms. NGA is a combined approach of genetic algorithm (GA) and neural network (NN) in which GA iterations are used for exploration and NN iterations for exploitation. Where there is no way to execute a communication schedule, TSCH actions occur predetermined in each time slot. NGA is used to gain maximum throughput and minimum delay in TSCH and is implemented in IoT. In huge networks, NGA is employed to maximize the turnaround time of successive node transmission and minimize the time slots. Moreover, to enhance the efficiency of the network, the correspondence nodes for concurrent transmission are scheduled in the optimal TSCH frame.

With the coexistence of graph and source routing, a heterogeneous routing model was proposed in [30]. In addition, a scheduling method which is called Relative-execution Deadline First (RDF) was proposed and validated against existing methods such as fixed priority (FP) and earliest deadline first (EDF) methods which showed better performance. A Mixed-Criticality Relative-execution Deadline scheduling algorithm (MCRD) was suggested to improve the system’s scheduling ability by considering a trade-off between reliability and real-time efficiency.

The main accomplishments of the mentioned paper are briefly discussed below:

1. A new heterogeneous routing containing source and graph routing was proposed to distinguish a flow’s criticality;
2. An RDF scheduling method was presented to control flow priorities for an unscheduled system;
3. By switching the system’s criticality mode to consider a trade-off between the performance of real-time and reliability, an MCRD algorithm was proposed by proposing the concept of mixed-criticality;
4. The acceptance ratio was effectively improved in the proposed performance evaluation method and validated versus several existing methods.

To verify IoT jobs formally and prepare a simulation environment for a typical RT-IoT application, a new cloud-based architecture where the possibility of real-time remote tasks was introduced [31].

Four important issues are the main accomplishments of this paper which are mentioned below:

- A cloud-centric tool to prevail the defects in common real-time systems was designed and implemented by activating remote access;
- Due to a maximum bound on traffic delays, an environment that can examine input IoT tasks under various scheduling policies was provided;
- Tracking and monitoring a scheduled job in a smart space without the need to go in place was provided.

Python is used for implementation for many reasons mentioned in the paper. The outcomes of the presented system are compared with the available advanced tools. Using IoT edge nodes of physical objects, a tool was developed to communicate with the Raspberry PI to which physical resources are connected, to control live simulation and analyze these physical tasks. In addition, communication delay was reviewed and compared among various IoT nodes utilizing widely used SDN-based tools such as ONOS and Mininet.

A new load scheduling algorithm optimized for real-time IoT applications in fog computing environments called Quantum Computing-inspired (QCi) was presented in [32]. Furthermore, to activate real-time service delivery, QCi-Neural Network Model was employed to specify the optimal computational node as a model for prediction.

The motivations of the mentioned research are as follows:

1. An optimal approach was introduced to schedule load in fog-based computing nodes over the fog computing platform;
2. A new QCi algorithm was introduced to allocate heterogeneous tasks for computation to the optimal fog node based on the Node Availability Index (NAI) parameter to gain optimal efficiency. The NAI is a factor for analyzing a fog-node for managing the current load in terms of computational power to be enabled;
3. To specify characteristics based on an optimal node, a predictive QCi-Neural Network Model was proposed, showing outstanding performance in real-time IoT application environments;
4. Validating the proposed method against other state-of-the-art algorithms on statistical analyzation, energy consumption, and task execution time to demonstrate the algorithm’s efficiency in a simulated complex environment in fog computing using the iFogSim simulator.

While the machines’ location is in industrial applications, a set of multicore computing nodes is implemented, which are interconnected, leading to closer communication and computation at the edge of the network called Fog Computing Platform (FCP). Mixed-criticality applications with safety criticality and time-dependent efficiency features simulated on the FCP are proposed in [33]. As mentioned in this paper, the FCP configuration greatly affects the control performance when virtualized control is implemented as tasks on FCP. So, the FCP configuration is considered an optimization problem and attempts to solve it with a proposed metaheuristic solution. Partitioning optimization, a more accurate measure of the quality-of-control, and a more realistic model of control applications are considered in their work compared with similar methods, evaluation of the proposed approach experiments on several test cases. Including temporal isolation for control applications, all the test cases gained balanced quality-of-control. At the same time, other state-of-the-art methods previously ignored some of the optimization criteria.

For mixed-criticality systems (MCS) in edge computing over 6G networks, a smart semi-partitioned scheduling strategy (SSPS) was proposed in [34]. To improve the system service quality, QoS is used in SSPS in addition to the acceptance rate of tasks and weighted schedulability. Considering service quality and timing on system tasks, a new method called smart semi-partitioned scheduling strategy (SSPS) was presented in [34] for MCS in edge computing of homogeneous multiprocessors over 6G networks. An efficient mechanism for facilitating LO-criticality tasks (jobs) is developed in SSPS to improve the QoS and the schedulability. At the same time, the system mode is changed from LO-criticality to HI-criticality. The proposed method consists of all processes of scheduling, including the processes of global and partitioned scheduling. SSPS was validated in experimental results against the existing states-of-the-arts algorithms in partition scheduling and gained the best performance.

#### Analyzed Smart Grid Applications

Table 6 summarizes the papers’ classification mentioned above and the key criteria to consider when evaluating the smart grid strategy in IoT applications. This method’s case studies include the following: Time SIoTted Channel Hopping (TSCH) scheduling, 3D printers real-time and manufacturing networks, fog environment, edge nodes inside a vehicle, eight applications in operation, set of tasks with low and high critically.

Table 7 outlines a method for evaluating the above research utilizing the assessment factors in IoT smart grid applications. Most research studies on the smart building method evaluated their proposed cost-cutting strategy, latency, throughput, effectiveness, execution time, and energy consumption characteristics.

### 4.4. Smart Home Application

Using the functionality of a mobile-based Short Message Service (SMS) application and microcontroller-based Arduino board, a new system for IoT-based smart home automation was proposed in [2]. To connect the automated home appliances and the Arduino module, Wi-Fi is employed. A new approach for real-time scheduling is recommended for controlling the home environment by introducing a new communication protocol and switching functionality. A priority-based real-time scheduling algorithm is suggested to control the smart home automation systems called Shortest Deadline First–Real-Time Task Scheduling (SDF–RTTS). The proposed method is based on the Arduino-based platform, independent of remoteness or the natural irrelevance of distance in controlling sensor-based technologies. So, irrespective of the distance between the appliance and the source, Short Message Service (SMS) can trigger the appliances. With focus on scheduling and decreasing the overall execution time, the network- and security-related issues are annoying in the mentioned work.

#### Analyzed Smart Home Applications

Table 8 summarizes the papers’ classification and the key criteria to consider when evaluating the smart home strategy in IoT applications. In this method, the most crucial context is providing a technique for assigning tasks in real-time utilizing the Arduino-based platform’s mobile SMS application.

Table 9 uses assessment factors in smart home IoT applications to describe an evaluation of the above study. The parameters listed below are execution time, the count of tasks, and the equipment. Most research studies on the smart home method have evaluated their offered approach in the execution time characteristics.

### 4.5. Smart Farming Application

A new architecture based on cloud was introduced in [35] to create an environment for the simulation of typical RT-IoT applications and verification of IoT jobs in formal form, considering the possibility of real-time remote tasks. As was claimed in this paper, the proposed tool supports the possible analysis of real-time tasks and puts out a real environment for monitoring and evaluating different IoT tasks in formal form for the first time. In addition, tracking and evaluating the real-time jobs scheduled in a smart space were performed in a centralized server. Accomplishments of this paper are listed below:

- A cloud-centric tool with remote access was designed and implemented to cross the problems in traditional real-time systems;
- Supposing communication delays at maximum and using several policies in scheduling, the feasibility analysis of incoming IoT tasks was provided in an environment;
- Without going on location, tracking and monitoring jobs scheduled in a smart space were provided in an environment.

The tool was communicated with Raspberry PI, which was physically connected to resources, to analyze the physical tasks.

#### Analyzed Smart Farming Applications

Table 10 summarizes the papers’ classification and the key criteria to consider when evaluating the smart farming strategy in IoT applications. In this method, the most crucial context is using the newest web-based platforms to simulate and display real-time task scheduling activity in a typical IoT environment, introducing innovative RT-IoT systems architecture.

Table 11 outlines a method for evaluating the above research utilizing the assessment factors in IoT smart farming applications. Most research studies on the smart farming method have evaluated their offered approach in the execution time characteristics.

### 4.6. Smart Building Application

To stimulate energy-aware manners in commercial buildings, a new framework was proposed, which is called IoT-based smartphone energy assistant (iSEA) [22].

iSEA method consists of three main parts:

- Individual occupants’ smartphone were tracked for energy-use behaviors;
- A supervised deep learning method was employed to identify their energy consumption;
- Personalized, tailored feedback was prepared to influence their consumption.

The iSEA uses an Energy Efficiency Index (EEI) through smartphone tracking to categorize their behaviors into inefficient and efficient categories. Four layers, including the communication, service, cloud, and physical layer, were proposed in the iSEA architecture.

The efficiency of the iSEA was validated using a test in a commercial building for 12 weeks with ten occupants to remove current limitations such as gathering and tracking personalized data and behavior and transferring individual appropriate information to personal occupants in a holistic fashion. The efficiency of the suggested method can be observed through results in increasing personalized energy-consume behaviors with a mean of 34% savings in energy. Moreover, as seen in the obtained results, commercial building occupants usually ignore controlling lighting systems when they leave a building, which causes waste energy. By using IoT sensors in commercial buildings, iSEA notably supports research efforts to measure and promote energy-aware behaviors at a minimal cost.

Small coverage and a power-aware model for fire detection based on IoT were introduced in smart cities [36] using several multi-functional sensors. The sleep scheduling method was used to reduce sensors’ energy consumption, which notably decreases energy consumption. So, extra nodes for continuously covering the target area are not a requirement. The proposed fire detection system features are mentioned below:

1. The network lifetime was maximized using partial coverage constraints and sleep-scheduling techniques;
2. The number of sensors was decreased;
3. Aggregated data from many sensors with fog computing was processed in real-time;
4. The accuracy of fire detection was validated with a real implemented Testbed.

A new interface was proposed to simulate an IoT fire detection model using fire, temperature, and gas sensors. A sleep-scheduling algorithm was employed to put the extra nodes into sleep mode based on the requirements of the required area coverage. In two parameters, such as network lifetime and count of the active nodes, the introduced method showed high efficiency compared with existing methods. The proposed method could apply to heterogeneous nodes by considering latency and data rate as QoS parameters. Furthermore, to improve the accuracy of detecting fire in smart cities, visual sensors (such as cameras) could add to the scalar sensors environment to work deeply on achieved images.

#### Analyzed Smart Building Applications

Table 12 summarizes the papers’ classification mentioned above and the key criteria to consider when evaluating the smart building strategy in IoT applications. In this method, the most crucial context is using the IoT to identify energy consumption in commercial buildings and using IoT to implement a fire alarm system in smart cities.

Table 13 outlines a method for evaluating the above research utilizing the assessment factors in IoT smart building applications. The parameters listed below are: estimated power loss, energy consumption, count of active nodes, and network lifetime in the smart building approach. Most research studies on the smart building method have evaluated their offered approach in the execution time characteristics.

### 4.7. Shopping System Applications

To construct an Internet of Manufacturing Things (IoMT), IoT was applied to car engine remanufacturing [26]. Identification technology was designed for any parts of a disassembled engine, and the re-manufacturable resources’ status was observed and supervised in real-time under the concept of IoMT. To decrease energy consumption and cost and dynamic management of remanufactured resources, a real-time scheduling algorithm for production and a model based on the information captured about remanufacturing was mathematically introduced. In addition, an optimization method based on Pareto was employed to gain the optimal solution.

The efficiency of the proposed method was confirmed in comparison with industrial solutions by simulation in the case of AER maintenance and reassembling. The remanufacturing energy consumption and cost were deducted by 34%, and were shown in simulation results where the worker load rate is balanced more.

To enhance the real-time decision-making capability in the scheduling system, a general architecture of distributed and flexible real-time workshop scheduling based on EC-IIoT was presented in [37]. This new method, called the DFJS-RS method, was designed and developed by combining two task assignment methods of the shop floor layer and the RS algorithm of the flexible manufacturing units (FMUs) layer. Moreover, a solver method was used based on an evolutionary game to achieve the optimal allocation. In four aspects, the main accomplishments of this paper could be categorized:

- By applying EC-IIoT to the DFJS, a general architecture of the DFJS-RS was introduced based on EC-IIoT, which can solve real-time decision-making problems in a shorter response time and deal with the data explosion;
- The RS method was executed to allocate operations to proper machines in real-time. Each time, the real-time allocating of the jobs was performed once, and initially, the scheduling was not generated by the RS method compared with the traditional rescheduling strategy. So, due to destroying the deviation between the original and new schedule, the production system works continuously and was more stable;
- For real-time allocating of the operations, and evolutionary allocation method based on the game was used. The proposed algorithm only assigns one operation each time to a machine compared with the other common algorithms where, by increasing the number of machines and jobs, the complexity of the method remains stable;
- The evolutionary game equilibrium method was used to find the best outcomes of the DFJS-RS problem to pass the traditional multi-objective optimization method’s disadvantages, such as specifying the weight coefficient in the weight algorithm and selecting feasible solutions in Pareto optimization.

A DFJS-RS case was used to analyze the efficiency of the suggested algorithm. Because of the three above reasons, the proposed method could not be evaluated in comparison with existing scheduling methods:

- Makespan;
- Critical machine workload;
- The energy consumption in production.

For reliable and fault-tolerant performance, collaborative scheduling of resources in energy-constrained applications was suggested in [38]. A task scheduling algorithm based on particle swarm optimization was proposed to allocate resources in cloud-based IoT applications (homogeneous and heterogeneous). They are called robust canonical particle swarm optimization (CPSO) and fully informed particle swarm optimization (FIPS) algorithms. The researchers’ goal was to fulfill the QoS in throughput and delay using optimal task scheduling by considering the several data gained through experience traffic categories. Considering the several experienced data traffic categories, delay and throughput as the QoS are considered objectives to accomplish optimal task scheduling. While the QoS is fully noticed, a metaheuristics scheduling algorithm is suggested for optimizing the management queue with the profit of the IoT resource allocation. Because converging to an optimal solution and simply searching are the main advantages of the PSO algorithm, it was used in the proposed method. The velocity of each particle was updated due to a distinct sensor/server kind in the PSO algorithm, which was used to serve the users’ requests. To distinct the priority difference between the low and high data traffic, the value of this velocity took constraints of QoS and the resource allocation policy into account. While an IoT application is requested, a queue system is assumed using some traffic flows to validate the suggested method depending on the QoS requirements. The service rate for each application and these requests are assumed to be Exponential and Poisson probability density function distribution, respectively. First in, first out (FIFO) is considered as the queue system. Moreover, for homogeneous and heterogeneous systems, for all servers, the service rates are equal and vary, respectively. Significant improvement in delay and throughput parameters as the quality-of-service parameters was shown in a simulation of the DDSS and h-DDSS architectures based on FIPS compared with CPSO.

#### Analyzed Shopping System Applications

Table 14 summarizes the papers’ classification mentioned above and the key criteria to consider when evaluating the shopping system strategy in IoT applications. In this method, the most crucial context was designing a validation method for isolated machinery pieces to monitor the state of recyclable resources in real-time.

Table 15 outlines a method for evaluating the above research utilizing the assessment factors in IoT shopping system applications. The parameters listed below are worker load rate, cost reduction, energy consumption, makespan, workload balance, delay, throughput, and QoS. Most research studies on the shopping system method evaluated their offered approach in the throughput, energy consumption, makespan, cost, and latency characteristics.

### 4.8. Energy Efficiency Applications

To arrange the leisure network devices effectively in the network edge and to organize integrated fog networks with the cloud, a new method was proposed to optimize the presentation of the massive amount of IoT services with considerable variety and provide computing resources and storage [24].

Two ILP models were proposed to overcome the integrated fog computing planning issue with the cloud (iCloudFog) framework. The objective of the first model is to decrease the cost of the CAPEX and the OPEX, which are created by planning fogs and using the planned fogs, respectively. The second one aims to lessen the power consumption while the count of favored prepared IoT tasks on the planned fogs is growing.

Several wireless and wired fog nodes were chosen as candidates in the fog layer. Then, some of these fog nodes were selected to optimize the overall performance in the iCloudFog and to optimize supply IoT tasks meeting their QoS requirements such as mobility and real time. The total cost is notably affected by the size of each fog and real-time and mobility as the QoS parameters as an observation of the numerical results where the power consumption is only affected by the QoS parameters. Although, in the process of fog planning, fully connected architecture is considered for planning fogs to find the optimal size of each fog as a case study.

A new method for task scheduling was proposed in [39] called Dynamic Fault-Tolerant Learning Automata (DFTLA). Tasks were allocated to the fog nodes efficiently in the proposed method using variable-structure learning automata.

The efficiency of the proposed DFTLA scheduler was evaluated in comparison with three benchmark methods which show better performance in robustness and reliability. In their future work, they proposed to hold a high-priority task on the fog node in the scheduling model since the process is incomplete. As an alternative solution to hold several high-priority tasks, neighbor nodes could be used to elude congestion in a certain point spot, while in some sections of the network, end-users are too many. Furthermore, the learning automata could consider the amount of remaining energy in fog nodes.

To solve the task scheduling in fog computing (TSFC) and to improve the quality of requirements by users, an energy-aware model using the marine predator’s algorithm (MPA) was proposed in two separate versions. To improve the exploitation strength, the last updated position is used instead of the previous best one in the first version of the proposed method, modified MPA (MMPA). Using re-initialization based on the strategy of mutation toward best and ranking, the latter improves MMPA. Although, the improved version of MMPA (IMMPA) shows outstanding results regarding flow time, makespan, carbon dioxide emission rate, and energy consumption by employing a genetic algorithm and some other meta-heuristic algorithms. Applying the proposed method for the DNA fragment assembly problem, scheduling the related tasks in the fog system, and dealing with the multi-dimensional knapsack problem were mentioned as future work [40].

In mobile cloud environments, a new method for multilevel trust improvement for effective task scheduling was proposed [41].

A trustworthy communication between two communicated nodes: MCC and mobile/IoT devices, was introduced in the proposed model.

The main accomplishments of the proposed method can be summarized as follows:

1. Trustable task scheduling was predicted to improve performance;
2. To interact with each other and swap trust boundaries, the task scheduler updates from trustor and trustee to make dynamic decisions through a trust computational algorithm;
3. Trust-based certification was used to run, and the dynamic trust manager selects only trusted tasks from trusted calculation models;
4. Trust evaluation and development are solved in the first algorithm, whereas in the second one, a new mobile node was added for evaluating trust. In the third and fourth algorithms, the task through effective decision-making efficiently offloads trustable tasks;
5. QoS was enhanced by using a multilevel algorithm in central trust management for scheduling tasks on IoT-based MCC;
6. In the simulation, mobile offloading was employed for the performance evaluation of the system. The results showed a significant improvement in the system decision-making and a decrease the power consumption. The problem of minimizing the total consumption of energy of IoT devices, as well as the weighted sum of AoI, was investigated in the article [42]. In the proposed model, all physical processes that follow nonlinear dynamics are monitored by every single IoT device. Since there is a timely change in the dynamics of the physical process, an optimal sampling frequency must be found by each device to sample the real-time dynamics of the physical system to send the sampled information to the base station. In the mentioned research, a new distributed reinforcement learning (RL) method for optimization of the sampling policy is suggested, which allows edge devices to select the globally optimal sampling policy using their sensing.

The problem of scheduling tasks for minimizing the mean energy consumption of fog nodes while considering the requirements of QoS of IoT tasks was mathematically formulated in [15]. Minimizing the violation time’s deadline was also considered in the model, and two semi-greedy algorithms, namely priority semi-greedy (PSG), and PSG with a multi-start procedure (PSG-M) were used to map IoT tasks to fog nodes efficiently.

Key accomplishments of the research could be seen as follows:

1. An MINLP model was proposed for IoT task scheduling in heterogeneous fog networks to optimize the energy consumption of fog nodes while considering task deadlines and minimizing the time of deadline violation;
2. To effectively map IoT tasks to existing fog nodes to provide high QoS for IoT users in terms of response time and to minimize the energy consumed by fog nodes, two semi-greedy-based algorithms named PSG and PSG-M are proposed;
3. The results showed that by using the presented algorithms, the deadline required by a big part of the tasks of the IoT is met, and the remaining small amount also provides its answer, they obtain some time violations. Considering that the total energy consumption and system lifetime are reduced, the benefit of service providers in fog is also increased.

It can be realized from the results that the suggested algorithms show significantly better performance regarding IoT tasks that meet the requirements of the deadline (in percent), the total consumption of energy and system lifetime, and the total rate of the violation time deadline than the existing algorithms. Using the suggested algorithms for scheduling dependent tasks in the IoT was suggested as future work [43].

With an average reward criterion, this online problem is formulated as an infinite-horizon-constrained Markov decision process (CMDP). Then, by employing the Lagrangian method and introducing a framework for the transformation of Lagrangian for the original CMDP problem, the CMDP problem is transformed into an unconstrained Markov decision process (MDP). Moreover, to gain the best policy for the CMDP problem, the proposed framework is integrated with a refinement mechanism based on the perturbation. According to the above challenges, the following key discussions are provided:

1. A novel measure, age of processing (AoP), is presented to obtain the novelty of the situation with respect to the processing of data in real-time IoT applications. The common mode sampling and the problem of offloading in the processing are formulated as an infinite-horizon Markov decision process (CMDP) with the maximum frequency of sampling constraint of the IoT device to minimize the average AoP;
2. Employing the Lagrangian algorithm to simplify the original problem of CMDP, the challenging CMDP problem is converted into an unconstrained MDP problem. A Lagrangian transform framework is also recommended to gather the optimal state sampling and policy of offloading in processing using the optimal Lagrangian multiplier;
3. Policy iteration algorithms based on stochastic approximation with perturbation-based refinement are developed to gain the best policy for the CMDP problem based on the proposed Lagrangian transform framework;
4. To demonstrate the structural features of the proposed optimal policy, extensive simulations were performed to show that the algorithm surpasses the benchmarks by reducing the mean AoP by 30%.

Generalizing the proposed framework to the highly challenging scenarios with multiple IoT devices and edge servers is suggested as future work.

#### Analyzed Energy Efficiency Applications

Table 16 summarizes the papers’ classification mentioned above and the key criteria to consider when evaluating the energy efficiency strategy in IoT applications.

Table 17 outlines a method for evaluating the above studies utilizing the assessment factors in IoT energy efficiency applications. Most research studies on the energy efficiency method evaluated their approach in the makespan, execution time, and cost characteristics.

### 4.9. Health Monitoring Applications

In the study by [11], an intelligent patient health monitoring system (PHMS) was presented for remote monitoring of patients’ vital signs data, which operates based on an optimal scheduling mechanism using IoT task coordination architecture. The two main modules of this system are:

1. Employing a real-time IoT-based task coordination architecture, an optimal time constraint-aware mechanism for scheduling was introduced to manage and extend autonomous and emergency healthcare tasks effectively;
2. An optimization module based on objective functions was introduced to optimize the electronic health industries’ services.

In the mentioned research, the Libelium e-Health toolkit was employed to monitor patient’s physiological data remotely.

Below are the significant accomplishments of this article:

- To effectively remotely monitor patients’ vital signs data, a mechanism based on time constraints using real-time tasks based on IoT coordination architecture and optimization scheduling techniques was introduced;
- To minimize the information lost when switching the context of the sensing devices and to reduce the ratio of the sensor miss to improve the reliability and recovery of the introduced intelligent PHMS, a module for optimization using the objective function was developed;
- A time-constraint-aware PSO-enabled scheduling strategy was introduced for efficiently scheduling healthcare tasks using a real-time architecture for task coordination based on IoT;
- A task coordination IoT-based architecture was presented to effectively and dynamically address the extension of critical and emergency healthcare tasks in real time;
- In the mentioned research, various evaluation criteria were utilized to examine the effectiveness of the proposed intelligent PHMS, such as starvation rate, throughput, RTT, latency, task drop rate, and response time.

In the paper by [12], it was claimed that the starvation and miss ratio in healthcare tasks to enhance the performance of the introduced intelligent PHMS can be reduced via the suggested method for an optimal scheduling mechanism. While the ratio of starvation of the introduced optimal scheduling method is 12%, this parameter in the FEF and RM task scheduling method is 26% and 28%, respectively, which shows a dramatic enhancement in the overall performance of the health care tasks of the proposed scheduling mechanism. Moreover, in comparison with the FEF and RM scheduling method, the hunger rate of the tasks of the proposed optimal scheduling method has decreased by 16% and 14%. Moreover, compared with the basic RM scheduling and FEF scheduling, the optimized scheduling method shows a task reduction rate of 21% and 17%. Assuming tasks with conditional limitation on Voltage Frequency Island (VFI)-based heterogeneous No C-MPSoCs and setup of rescheduling integrated with DVFS for real-time streaming applications, a new energy-aware scheduler was proposed. A novel task-level rescheduling approach called R-CTG was integrated with scheduling based on non-linear programming, and a method for scaling voltage regarding ALI-EBAD was proposed. The proposed method’s aim was to minimize the latency caused by rescheduling without dealing with energy efficiency. Because of releasing the wasted slack in re-times tasks in the proposed method, significantly reduced rescheduling latency was reported compared with the state-of-the-art algorithms developed for traditional tasks based on Directed Acyclic Graph (DAG).

As an optimization problem the study by [44], the scheduling of IoT service requests was modeled using integer programming to minimize the overall service request latency. Naturally, the scheduling problem is NP-hard and cannot be solved by exact solutions for optimization in large-size problems. So, for scheduling the IoT requests, a reconstructed implementation of the genetic algorithm (GA) was employed for overall minimizing the latency. In a dynamic simulation environment, the performance of the proposed method is estimated in comparison with several known methods, such as priority-strict queuing (PSQ), round robin (RR) algorithm, and waited-fair queuing (WFQ). Modeling the problem of assigning scheduling IoT requests produced by edge devices to the sufficient availability of resources in the fog and cloud is the main goal of this research. The ILP technique and a customized GA are employed to gain the minimum service time for IoT requests and a possible solution with the best in-hand quality in an acceptable calculation time by using the possible gaps in the scheduling of cloud and fog VMs, the main goal of the research by [13] was to schedule computing tasks with low needs for communication in the cloud and centralized tasks for communication with low computing needs in the fog. In addition, the introduced approach considers the costs of communication caused by the data transmission between sensors in the IoT layer and the devices in the fog layer during the scheduling process. By simulating with a base cloud-unaware strategy and using several workload cases, the performance evaluation of the introduced algorithm is analyzed and compared with states-of-the-arts algorithms. In an architecture with three layers, a hybrid cloud and fog-aware heuristic was proposed for dynamic real-time scheduling of multiple IoT workflows. In this approach, an attempt was made to use possible gaps in the cloud and fog VMs. Unlike traditional methods where the principal processing of IoT tasks is executed in the fog layer, computing tasks with low needs for communication in the cloud and centralized tasks for communication with low computing needs in the fog can be scheduled. In the study, the cost of communication caused by the data transmission between devices and sensors in the IoT layer and VMs in the fog layer is considered in the scheduling of VMs.

A resource-aware scheduler called Resource-Aware Cost-Efficient Scheduler (RACE) was proposed to broadcast the forthcoming application modules to fog devices [45]. The RACE algorithm reduces the pocket cost of utilizing the resources in the cloud with a minimum time of execution of applications and minimum bandwidth usage. Moreover, it maximizes the utilization of resources at the fog layer. Due to the requirements of their bandwidth and calculations, forthcoming application modules are categorized. Simulation analysis using the iFogSim simulator shows better efficiency of the introduced method compared with the traditional cloud assignment and the base algorithm in most of the parameters.

The accomplishment of this research could categorize as follows:

- To maximize resource utilization, the RACE scheduler was proposed to assign application modules from the fog layer to the cloud layer instead of transmitting them to the cloud directly;
- For minimizing time of execution, pocket cost, and used bandwidth, of using cloud resources;
- A priority mechanism was proposed to assign application modules compared with assignment methods in the traditional cloud and cloud–fog from several viewpoints; using resource-aware policy in simulating fog environment in the iFogSim gained considerable improvement.

In terms of lowest bandwidth, least monetary cost, and minimum execution time in three different strategies (direct assignment of the workload on the cloud, workload assignment on the fog layer, and workload assignment from the fog layer to the cloud layer), the results of the evaluation of the RACE algorithm are excellent. While the RACE(CFP) has better performance in the overall performance in monetary cost, execution time, and bandwidth consumption, in some cases in pocket cost when fog devices’ numbers are bigger, the RACE(FOP) efficiency is better.

#### Analyzed Health Monitoring Applications

Table 18 summarizes the papers’ classification mentioned above and the key criteria to consider when evaluating the health monitoring strategy in IoT applications. This method’s case studies include the following: healthcare, 12 real benchmarks, fog and cloud, a three-tiered structure for IoT processes, and three different network topologies with other workloads.

Table 19 outlines a method for evaluating the above research utilizing the assessment factors in IoT health monitoring applications. Most research studies in the health monitoring method have evaluated their offered approach in the makespan, execution time, cost, latency, and energy consumption characteristics.

### 4.10. Other Application Areas

A task scheduling policy based on RL was presented to execute the tasks in the minimum possible time, optimally use the resources, and dramatically reduce communication costs during the distributed execution. A policy for task scheduling was presented based on RL [46].

Proposing this task scheduling system using a two-level artificial neural network (ANN) can determine whether a data stream can be processed in an environment that is constrained by resources (edge/fog) and be analyzed (executed) or should be sent directly to the cloud by the first-level ANN (Feedback Neural Network/Convolutional Neural Network (CNN)).

Ergo, the task of the 2nd NN level (RL module) is to schedule, among the available fog devices, all the functions sent by the 1st NN level to the fog layer. This policy for real-time task assignment was used to reduce communication costs and the total computational delay (makespan). Combining RL-based task scheduling with the task clustering algorithm minimizes communication costs with the benefit of scheduling the whole cluster on one computational resource.

To perform the analysis for the task assignment employing five fog devices was taken into action. The number of instructions performed every second on that fog device is computed capacity. The DAG makespan in the RL-based scheduling policy is the least compared with a greedy algorithm; thus, the RL-based scheduling utilizes resources better.

The purpose of this research aimed by its researchers was to present a novel variant of the optimistic concurrency control protocol. Partially deployed augmented validation protocol can make it possible to locally process the read-only IoT transactions at the fog node [16].

Only updated transactions are sent to the cloud to reach the ultimate validity. Furthermore, the updated transactions can have more chance to commit to the cloud if they pass through a partial guarantee at the fog node. This protocol minimizes computation and communication in the cloud while maintaining the capacity to support transactional services utilized by applications running in such environments.

The paper aimed to highlight the realistic running workload managed by the updated read-only transactions. At the transactions of IoT applications, partial validation is used to immediately find conflicts to save resources and avoid delays in communication. The algorithm used to validate transactions received from the fog nodes is sophisticated. The purpose of the researchers of this study is to present an approach for processing transactions in these environments. Via a new protocol, a part of the function for validating with the server should be shared by the IoT clients, and they should be capable of detecting data conflicts early. This unique protocol is believed to be more beneficial for IoT-based environments. Via the framework for transaction processing, it can be determined by the client if active transactions can be canceled or continued. It could be specified as selected while the processes of the transactions are implemented at the server based on the serializable conflict researcher assessed the function of partial validation using three concurrent protocols, which are based on numerical survey protocols. The study’s results showed that using the suggested algorithm should be helpful for IoT users. This can be obtained from transactional services.

In conclusion, the assessment showed the theory that the high abort rates problem presented by the optimistic concurrent control shall be reduced significantly via caching. The issue of the abort rate is rectified via the introduced optimizations, i.e., a validation prepared partially for the local performing of writing operations at the fog and read-only transactions. Several key observations are gained through different experiments that have shown that partial validation is suitable for supporting applications based on IoT data transactions.

In the study by [47], an extended particle swarm optimization (EPSO) algorithm was introduced using different gradient methods for optimizing task scheduling in cloud–fog environments. The first important goal is to enhance the resources’ performance and minimize the time needed to complete a task. The study mainly focused on developing a new model based on proximal gradient PSO algorithm for optimizing task scheduling in cloud–fog environments. It provides a new model by which the performance of the resources can be improved; the completion time can be minimized.

In recent years, CC platforms have been broadly employed and evaluated in the CloudSim; thus, fog computing is evaluated on the iFogSim to support services and simulation of architecture in a cloud–fog environment. For validation of the performance of the EPSO algorithm in comparison with the other techniques, two parameters were used: makespan and total cost. The experiments were performed on seven simulated tasks regarding the total cost and makespan. Then they were compared for the efficiency of the proposed EPSO algorithm in comparison with MPSO, TCS, BLA, and ideal PSO.

It can be understood through the results that a makespan total cost gained by the introduced EPSO algorithm is much more economical than the one achieved through MPSO. Requirements of the end-user in terms of high processing performance and cost efficiency were also fulfilled by EPSO in a better way. It seemed that performing a huge number of tasks on fog nodes can help decrease the delay time; however, it can enhance the energy consumption at the level of the fog node. For making a trade-off between energy consumption and delay, a strategy is devised using parallel meta-heuristic algorithms.

The proposed EPSO method was also compared with other traditional techniques, e.g., PSO and modified PSO, for their performances. It was understood at the end that the makespan achieved by EPSO and its performance is comparable with that of other approaches.

In this context [48], management and allocation of resources in an IoT-enabled CC environment are the issues addressed in the research by modeling a new quasi-oppositional Aquila optimizer-based task scheduling (QOAO-TS) technique. This technique integrates Aquila optimizer with the learning based on the quasi-oppositional. The common Aquila optimizer is motivated by the behavior of Aquila during prey capture, and the QOAO was developed to improve the Aquila optimizer’s efficiency. The objective of the QOAO-TS technique is to perform the makespan by optimal task scheduling. The proposed QOAO-TS technique considers the inter-scheduling relationship and fulfills the customer’s requirements within the shortest time interval. After performing many simulations, the results are checked and evaluated in terms of flow time, throughput, span, and the ratio of reduction and utilization.

A new algorithm for task scheduling was proposed using the Aquila optimizer-based task scheduling (QOAOTS) quasi-adversarial techniques in an IoT cloud environment. The QOAO technique incorporates quasi-adversarial-based learning (QOBL) with an aqueous optimizer to increase the efficiency of the Aquila optimizer. The objective of the QOAO-TS technique is to perform the required time by performing the optimal task scheduling process. In addition, the QOAO-TS technique has taken advantage of the relativity among task scheduling by minimizing the time interval and meeting the customer’s requirement. A detailed comparative analysis of the methods was performed to demonstrate the enhanced efficiency of the QOAO-TS technique over other available methods.

Detailed analysis methods for comparison are performed to show the enhanced performance of the QOAO-TS technique in comparison with the other state-of-the-art methods. The results of the QOAO-TS technique were compared with recent methods in several evaluation criteria. The combination of two meta-heuristic algorithms and deep learning methods can be introduced in the future for optimal scheduling of tasks in the cloud environment of IoT.

The study by [18] tested the coordination of real-time workflows in IoT in a heterogeneous environment in fog computing, using partial calculations of a partial analysis propagation mod and a dynamic scheduling heuristic in this context with end-to-end error propagation. While a workflow task generates an inaccurate result, the error may propagate both to its parent task and subsequent workflow tasks, ultimately affecting its development.

A comparison between the proposed scheduling algorithm and a benchmark algorithm was made, where partial calculations were not employed, using several thresholds and probabilities for result precision and propagation of input error. The proposed algorithm for scheduling also focuses on the influence of the initial IoT input data transfer from the IoT layer to the fog layer. It was validated with a benchmark algorithm, where partial calculations are not employed, using different thresholds of result accuracy and input error propagation probabilities.

Makespan: using analytical solutions, it is required to simplify assumptions which finally lead to illusory results. 

iFogSim workload: the simulation analysis showed that the introduced heuristic worked better than the baseline policy regarding the ratio of deadline misses for a small loss in the quality of outcomes. It can be specifically realized and observed that the average deadline miss ratio decrease via PC was better than the baseline policy; however, the mean reduction in the outcomes’ precision was not satisfactory enough. Moreover, the simulation outcomes have exhibited that the introduced method was flexible to the effects of input error diffusion among the workflow’s tasks.

The authors of [49] considered the problem of task scheduling in the EC sc. At the edge server, many tasks are assigned to VMs configured by increasing the long-term task satisfaction degree (LTSD). Markov Decision Process (MDP) formula was used for the problem with various related designs, e.g., state, action, state transition, and reward. Deep reinforcement learning (DRL) was leveraged to solve both time scheduling and resource allocation, of which examples are task execution order and VM tasks assignments, respectively. Diverse functions of the studies and heterogeneity of existing resources are considered. An RL algorithm based on a policy was introduced for the problem of scheduling the task, and a fully connected neural network (FCN) was used for feature extraction. RL is a proper method developed to handle MDP problems. In this approach, the agent continuously makes sequential decisions using the interaction with the environment.

The article focused on task scheduling based on the DRL to be considered for scheduling tasks in edge computing. In this model, the assignment of VM and time schedules are optimized together. MDP is the formulation where VMs availability, task features, and dynamic queues are considered.

Another main issue this article considered as one of the main points is the action presented as a VM-task pair with large dimensions. The MDP formulation mechanism is updated, and the time scheduling step is separated from the real-time time step. Thus, the action space remains linear with the number of VMs and queue size, and many tasks could be scheduled together.

The research by [50] attempted to find the incorporation between the Chimp optimizing algorithm (ChOA) and Marine Predator’s Algorithm (MPA) as well as the operator of the disruption to specify the optimal solution for scheduling IoT applications’ task. The proposed algorithm (CHMPAD) aims to avoid getting stuck in local minima and enhance the capability of exploiting traditional ChOA as its main disadvantage. The main purpose of this research that should be paid enough attention to is proposing an intelligent task scheduling method employing an enhanced ChOA based on DO and MPA for IoT applications in fog computing environments. Another purpose is to consider measures such as the ratio of performance enhancement, throughput time, and the makespan to affirm the QoS requirements of IoT devices. Finally, the efficiency of the introduced CHMPAD algorithm was validated using the evaluation parameters by performing several tests with several different tasks employing a general CloudSim toolkit platform. To check whether the development of CHMPAD was performed effectively, the efficiency evaluations of the algorithm were experimented using the CloudSim toolkit selected for the tests in the study since it supports modeling and simulates complicated and large calculation environments.

To sum, it can be noticed that the reported experimental results in the research show a great enhancement in the throughput and makespan time on most of the criterion instances. Furthermore, it can also be understood via results that the approach proposed in the study can boost computing’s throughput. It is already planned to implement the CHMPAD scheduling algorithm in a real natural fog computing system. To reach a suitable solution for more complex optimization problems such as planning health care facilities, quadratic assignment, assembly line balancing, and vehicle routing, CHMPAD may be improved as well.

#### Analyzed Other Application Areas

Table 20 summarizes the papers’ classification mentioned above and the key criteria to consider when evaluating the other areas in IoT applications.

Table 21 outlines a method for evaluating the above research utilizing the assessment factors in IoT and other application areas. Most research studies in the different areas evaluated their offered approach in the makespan, cost, throughput, and execution time characteristics.

## 5. Discussion and Comparison

The previous sections explained how to review selected articles on real-time scheduling in the IoT. This section presents a statistical analysis of what was stated in the previous sections. Moreover, in response to AQ3, statistical reports are presented as follows:

- AQ1: What are the practical areas of real-time scheduling in IoT?

**Figure 5:**
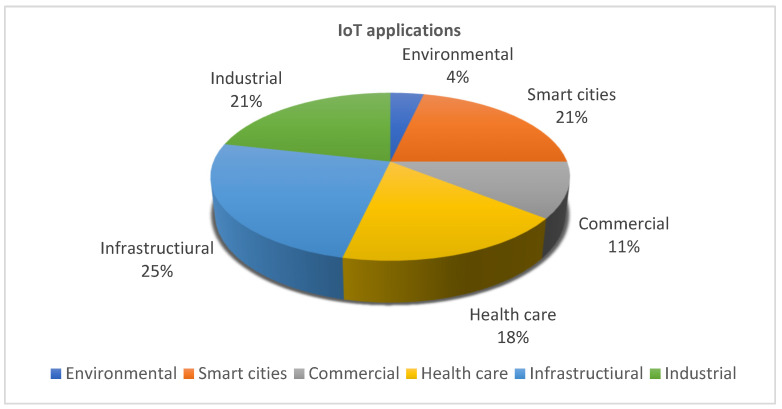
Amount of IoT applications in different spaces.

Figure 5 shows the percentage of practical applications of the IoT, and Section 4 explains this in more depth. The six main applications of the IoT include healthcare, the environment, commerce, the smart city, industry, and infrastructure. Each has sub-sections and sub-sections, as shown in the previous figure. According to the reviewed articles, the infrastructure has the highest percentage of application domains, with 25%. Industrial and smart city applications each account for 21%, healthcare applications for 18%, business applications for 11%, and environmental applications for 4%.

- AQ2: How are real-time schedulings in the IoT evaluated?

**Figure 6:**
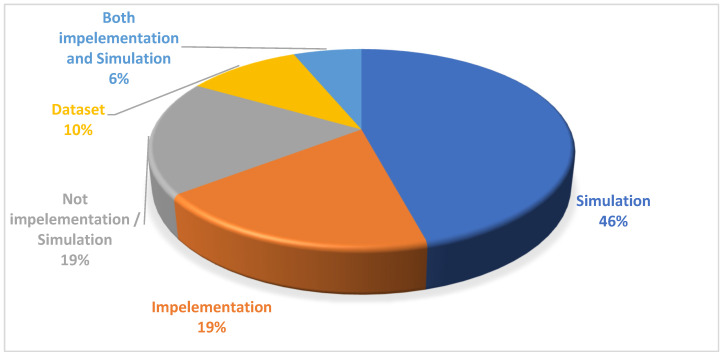
Evaluation environments are used.

According to the percentages in Figure 6, 19% of the articles proved their point of view through implementation. We also see that 46% of the articles used simulation tools to evaluate the studies presented. In addition, 19% of the articles lacked implementation or simulation for their work, while 6% included both. Finally, it is observed that 10% of the present articles used datasets to evaluate the articles.

- AQ3: What are the most prevalent criteria for IoT real-time scheduling evaluation?

**Figure 7:**
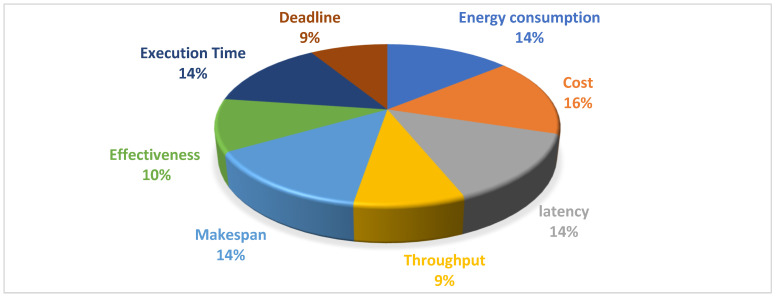
Evaluation parameters of real-time scheduling in IoT applications.

The percentage of evaluation parameters in selected articles is shown in Figure 7. Based on the specified values, the cost has the highest percentage in evaluating applied approaches with 16%, energy consumption, construction time, execution time, and delay of 14% each, the effectiveness of 10%, deadline, and operational capacity of 9% each.

### Future Work and Open Perspectives

Given the application of the SLR process to the IoT application suite, future work and open perspectives are provided as AQ4 for the following research problems.

- AQ4: What are the future directions and open perspectives for IoT applications?
- Network performance: Network performance is crucial in IoT development. By considering the influence of different fog topologies (rings, stars, etc.) and heterogeneous nodes with respect to latency and data rates, network performance is improved. For example, by adding visual sensors (cameras) to the sensors, fire detection becomes much more accurate;
- Residual energy from the node: before assigning a task to a node for processing, it is checked how much energy is needed to process that task;
- Improving the performance of health care services: improving health care services by predicting the patient’s vital signs and prognosis with the IoT;
- Communication costs: The data generated from the sensors of IoT sensors are transferred to VMs for scheduling and processing, and this process includes costs called communication costs. By scrutinizing these costs more closely, the scheduling process improves;
- Maximize resource utilization: Use additional cloud resources to schedule IoT workflows in cases of increased workload. Balancing cloud and fog computing resources through intermediate tools increases resource usage. This balance creates effective cooperation between the cloud and the edge. Moreover, by using objective functions, the use of resources is maximized, and latency is minimized;
- Implementation of scheduling algorithms in real fog computing system: by expanding scheduling algorithms in the real fog computing system, overcoming optimization problems, vehicle navigation, and improving the quality of health care is provided;
- The reliability, and real-time execution of the task are improved by adding a new computing node to the network;
- Improve network security: network security is improved by correctly checking network bandwidth and allocating network resources.

## 6. Conclusions

Real-time IoT scheduling publications were analyzed in this study using a systematic review method, and the overall concept of IoT real-time scheduling, aims, and open issues were determined. There are 162 articles in this review article. The items in this collection were published between 2018 and 2020. Finally, we looked at 35 publications about IoT scheduling in real-time. The practical application of industrial energy efficiency has the greatest percentage of implemented approaches in this study, with a 20% quota. Of course, industrial smart grid applications account for 17%; healthcare, 14%; commercial sales systems, 8%; smart buildings and urban with 6% each; traffic monitoring, smart homes, and smart farms for 3% each; and other aspects of IoT applications account for 20% of the areas in IoT, according to AQ1. According to AQ2, 46% of articles used simulation tools to evaluate their proposed real-time Internet scheduling options. A total of 19% of the selected articles used the implementation, 19% did not present any simulation or implementation, and only 6% combined simulation and implementation. Furthermore, the dataset was used by 10% of the current authors to evaluate their proposed methodologies. In response to AQ3, it was discovered that the cost had the highest rate of real-time scheduling in the IoT at 19%. The next chairs had 17% delay, 16% runtime, 16% makespan, 14% energy consumption, 12% efficiency, 10% capacity, and 10% deadline. Because of the systematic overview of publications, not all articles were reviewed, and assessment articles, conference articles with few citations, and book chapters were eliminated. We conducted a complete study of real-time scheduling in this assessment piece by analyzing the content of 162 articles. As a result, given the growing number of research articles on this topic, it is apparent that not all articles become reviewed, despite this article being written in 2022.

## Figures and Tables

**Table 1:** Description of some evaluation factors used in the article.

Evaluation Actor	Definition/Description
Latency	The time it takes for data to pass from one point on a network to another [11].
Energy consumption	Using energy suitably and sufficiently with the least energy loss [12].
Cost	The cost of reserved dedicated hosts in the cloud layer and file transfer into or out of the cloud in the considered period [13].
Throughput	The production rate at which something is processed [14].
Makespan	The total time required to complete all tasks [15].
Effectiveness	The degree to which something is successful in producing the desired result; success [16].
Execution time	The final stage of a computer program’s life cycle is in which the code is being performed on the computer’s central processing unit as machine code [17].
Deadline	A task must be completed within the specified time frame [18].

**Table 2:** Categorizing the state-of-the-art in urban computing applications.

Study	Attributes
Guevara et al. [17]	Main concept: Providing a scheduling algorithm to minimize the length of cloud computing applications.
	Case study: Cloud–fog system.
	App domain: Cellular network/5G fog network.
	Advantage: Quick execution of tasks taking into account QoS.
	Disadvantage: Lack of attention to the cost of resources.
	Simulation/Implementation: Implemented in Java.
	Dataset: -
	Future work: Use CASSIA-RR for cloud-cloud systems where quick decisions need to be made.
Kaur et al. [28]	Main concept: Providing an algorithm in a multi-layer fog network with different job profiles that schedule real-time tasks.
	Case study: Fog nodes.
	App domain: Fog networks.
	Advantage: Avoiding delay and completion of tasks before the deadline.
	Disadvantage: -
	Simulation/Implementation: Used the iFogSim simulator.
	Dataset: -
	Future work: extending performance in real-time scheduling in multilayer architectures.

**Table 3:** Analysis of articles in terms of evaluation parameters in urban computing applications.

Study	Deadline	Cost	Latency	Throughput	Effectiveness	Execution Time	Makespan	Energy Consumption
Guevara et al. [17]	✗	✗	✗	✗	✓	✓	✓	✗
Kaur et al. [28]	✗	✓	✗	✓	✓	✗	✗	✗

**Table 4:** Categorizing the state-of-the-art in Traffic Monitoring applications.

Study	Attributes
Zhang et al. [29]	Main concept: For humans and automobiles, real-time traffic light scheduling.
	Case study: (1) Analysis of a simplified traffic network profile using weights; (2) a bidirectional four-way pedestrian-vehicle mixed-flow network.
	App domain: Traffic network.
	Advantage: Reduced traffic costs.
	Disadvantage: Tolerable reduction in performance
	simulated and implemented using Vissim and Matlab.
	Dataset: -
	Future work: Development of relevant model identification algorithms for estimating pedestrian flow rate.

**Table 5:** Analysis of articles in terms of evaluation parameters in traffic monitoring applications.

Study	Deadline	Cost	Latency	Throughput	Effectiveness	Execution Time	Makespan	Energy Consumption
Zhang et al. [29]	✗	✓	✗	✗	✗	✗	✗	✗

**Table 6:** Categorizing the state-of-the-art in smart grid applications.

Study	Attributes
Pavithra et al. [14]	Main concept: Providing an algorithm to minimize latency and maximize TSCH throughput,
	Case study:
	App domain: Wireless module and RF.
	Advantage: Improving- and comprehensively scheduling the TSCH to prevent network interference.
	Disadvantage: Costs were not considered.
	Simulation/Implementation: The experiment runs under a multichannel TDMA protocol.
	Dataset: -
	Future work: -
Xia et al. [30]	Main concept: Introducing the concept of a hybrid critical system to IIoT to balance real-time and reliability.
	Case study: -
	App domain: Industrial Internet of Things (IIoT).
	Advantage: Improving task scheduling by prioritizing based on relative execution deadlines.
	Disadvantage: Cost not considered.
	Simulation/Implementation: -
	Dataset: -
	Future work: By placing edge computing nodes on the network’s edge, you can improve the network’s dependability and real-time performance. Industrial wireless networks are also evaluated for secure transmission.
Darwish et al. [31]	Main concept: A real-time green-aware multiplex scheduling framework for customized 3DPTs in the IIoT is proposed.
	Case study: -
	App domain: IoT in the Industrial Sector (IIoT).
	Advantage: Improving energy efficiency and resource usage, which helps the economic and political pillars of sustainability.
	Disadvantage: -
	Simulation/Implementation: MATLAB® R2019b is used to run the simulations.
	Dataset: -
	Future work: Incorporating additional algorithms, such as game theory, into the suggested algorithm.
Bhatia et al. [32]	Main concept: Real-time optimization of fog-based scheduling.
	Case study: Fog environment.
	App domain: QCi-Neural Network.
	Advantage: It predicts the best node for fog computing platforms in a very efficient and real-time manner.
	Disadvantage: -
	Simulation/Implementation: Using iFogSim simulation toolkit.
	Dataset: Dataset is made up of heterogeneous data with varying loads, such as picture, movie, and audio signals.
	Future work: Security and trustworthiness for distributed environments and network bandwidth and resource allocation.
Barzegaran et al. [33]	Main concept: Determine how tasks are assigned to sectors, partition scheduling tables, and task scheduling tables, as well as how tasks are assigned to the core of edge devices.
	Case study: -
	App domain: Fog Computing Platform (FCP).
	Advantage: Maximize quality of control with FCP configuration.
	Disadvantage: Static cyclic scheduling.
	Simulation/Implementation: Simulation and C# implementation.
	Dataset: -
	Future work: Consider the effect of communication and other optimization strategies in solving the problem, as well as the feasibility of progressive scheduling based on the selected methodology.
Wang et al. [34]	Main concept: Scheduling Mixed Critical Systems (MCS) in a 6G-based edge computing environment on a multiprocessing platform.
	Case study: -
	App domain: 6G wireless communication.
	Advantage: SSPS not only completes critical HI tasks, but also selects critical LO tasks to execute.
	Disadvantage: Heterogeneous processors are not used.
	Simulation/Implementation: Experiments were run on a PC with a quad-core processor running at 3.40 GHz and 8 GB of RAM.
	Dataset: -
	Future work: Using the recommended SSPS algorithm to optimize multi-path parallel data transfer mechanisms for the IoT.

**Table 7:** Analysis of articles in terms of evaluation parameters in smart grid applications.

Study	Deadline	Cost	Latency	Throughput	Effectiveness	Execution Time	Makespan	Energy Consumption
Pavithra et al. [14]	✗	✗	✓	✓	✗	✗	✗	✗
Xia et al. [30]	✗	✗	✗	✗	✓	✓	✗	✗
Darwish et al. [31]	✗	✗	✗	✗	✓	✓	✗	✗
Bhatia et al. [32]	✗	✗	✗	✗	✓	✓	✗	✓
Barzegaran et al. [33]	✗	✓	✓	✗	✓	✗	✗	✗
Wang et al. [34]	✗	✗	✗	✗	✓	✗	✗	✗

**Table 8:** Categorizing the state-of-the-art in smart home applications.

Study	Attributes
Bhattacharyya et al. [2]	Main concept: Provide a technique for assigning tasks in real time utilizing the Arduino-based platform’s mobile SMS application.
	Case study: -
	App domain: Wi-Fi connectivity.
	Advantage: Minimize overall execution time.
	Disadvantage: Security- and network-related issues were not considered.
	Simulation/Implementation: The experiment used GSM SIM 300 and Arduino.
	Dataset: -
	Future work: But in future work, will focus on network and security issues.

**Table 9:** Analysis of articles in terms of evaluation parameters in smart home applications.

Study	Deadline	Cost	Latency	Throughput	Effectiveness	Execution Time	Makespan	Energy Consumption
Bhattacharyya et al. [2]	✗	✗	✗	✗	✗	✓	✗	✗

**Table 10:** Categorizing the state-of-the-art in smart farming applications.

Study	Attributes
Ahmad et al. [35]	Main concept: Using the newest web-based platforms to simulate and display real-time scheduling of tasks activity in a typical IoT environment, introduce innovative architecture in RT-IoT systems.
	Case study: -
	App domain: Web technologies (web-based tool).
	Advantage: Ability to instantly track and evaluate IoT tasks in real time via remote access to the system.
	Disadvantage: -
	Simulation/Implementation: Use Python3.
	Dataset: -
	Future work: In the future, the suggested tool can be improved and used in a real scenario in a smart home with real actuators and sensors to track more important metrics such as energy and power consumption, as well as to calculate the latency of actual communication profiles based on real-world interactions.

**Table 11:** Analysis of articles in terms of evaluation parameters in smart farming applications.

Study	Deadline	Cost	Latency	Throughput	Effectiveness	Execution Time	Makespan	Energy Consumption
Ahmad et al. [35]	✗	✗	✗	✗	✗	✓	✗	✗

**Table 12:** Categorizing the state-of-the-art in smart building applications.

Study	Attributes
Rafsanjani et al. [22]	Main concept: Using the IoT to identify energy consumption in commercial buildings.
	Case study: -
	App domain: Wi-Fi network.
	Advantage: Improving energy consumption behavior.
	Disadvantage: Energy-saving behaviors are observed only during the test period.
	Simulation/Implementation: The experiment used Python.
	Dataset: -
	Future work: Through iSEA performance in commercial building staging areas, evaluate the iSEA front-end system (the service layer).
	Main concept: Using IoT to implement a fire alarm system in smart cities.
El-Hosseini et al. [36]	Case study: -
	App domain: Wireless sensor network (WSN).
	Advantage: (1) Saving consumed energy; (2) Reducing the number of sensor nodes; (3) Increasing the network’s lifespan.
	Disadvantage: Lack of attention to delays and data rates.
	Simulation/Implementation: MATLAB 2014 is used to carry out the simulation. An Arduino and a Raspberry Pi were used in the tests.
	Dataset: -
	Future work: (1) Use the provided technique with heterogeneous nodes while considering delay and data rate. (2) Combine visual sensors (cameras) with the test bed’s scalar sensors to improve fire detection accuracy in smart cities.

**Table 13:** Analysis of articles in terms of evaluation parameters in smart building applications.

Study	Deadline	Cost	Latency	Throughput	Effectiveness	Execution Time	Makespan	Energy Consumption
Rafsanjani et al. [22]	✗	✗	✗	✗	✗	✗	✗	✗
El-Hosseini et al. [36]	✗	✗	✗	✗	✗	✓	✗	✗

**Table 14:** Categorizing the state-of-the-art in shopping system applications.

Study	Attributes
Zhang et al. [26]	Main concept: Designing a validation method for isolated machinery pieces to monitor the state of recyclable resources in real time.
	Case study: Reassembly and maintenance of AER.
	App domain: Domain of worker load, energy area, and cost area.
	Advantage: The remanufacturing sector, in particular for AER, is developing sustainably and producing cleaner products.
	Disadvantage: -
	Simulation/Implementation: -
	Dataset: -
	Future work: Concentrate on the mathematical-physical interaction between AS and MS models, as well as improve the approach for solving the RTPS challenge with more goals and realistic restrictions.
Wang et al. [37]	Main concept: Provide a comprehensive EC-IIoT architecture to improve real-time scheduling capabilities.
	Case study: Collaboration company in Xi’an.
	App domain: Edge computing.
	Advantage: Establishing the framework of the EC-IIoT-based DFJS-RS, which enables the capture and treatment of massive amounts of data quickly and efficiently.
	Disadvantage: The scarcity of case studies.
	Simulation/Implementation: -
	Dataset: -
	Future work: By placing edge computing nodes on the network’s edge, you can improve the network’s dependability and real-time performance. Industrial wireless networks will also be evaluated for secure transmission.
Al-Turjman et al. [38]	Main concept: Simple search and convergence to the optimal solution.
	Case study: a cloud environment.
	App domain: Wireless area network (WAN), 3G, and 4G networks.
	Advantage: High-quality services in terms of throughput and low latency.
	Disadvantage: -
	Simulation/Implementation: -
	Dataset: -
	Future work: The suggested system can operate as a middleware among cloud and edge computing resources, allowing for efficient IoT task scheduling collaboration between the edge and the cloud.

**Table 15:** Analysis of articles in terms of evaluation parameters in shopping system applications.

Study	Deadline	Cost	Latency	Throughput	Effectiveness	Execution Time	Makespan	Energy Consumption
Zhang et al. [26]	✗	✓	✗	✗	✗	✗	✗	✓
Wang et al. [37]	✗	✗	✗	✗	✗	✓	✓	✗
Al-Turjman et al. [38]	✗	✗	✓	✓	✗	✗	✗	✗

**Table 16:** Categorizing the state-of-the-art in energy efficiency applications.

Study	Attributes
He et al. [24]	Main concept: Introducing two ILP models that attempt to reduce total cost and energy usage without requiring real-time calculations.
	Case study: -
	App domain: Wireless fog (WLF); Wired fog (WDF); Hybrid fog (HBF).
	Advantage: Minimize total cost and energy consumption when performing IoT tasks.
	Disadvantage: -
	Simulation/Implementation: The AMPL model language and the Gurobi 8.1.0 solver were utilized in the simulation.
	Dataset: -
	Future work: Evaluating the influence of various fog topologies, such as ring, star, and others on the iCloudFog framework’s network quality.
Ghanavati et al. [39]	Main concept: Provide a new work scheduling approach with dynamic fault tolerance. (DFTLA)
	Case study: -
	App domain: Wireless connections-wide-area network (WAN).
	Advantage: Efficient summary execution of heterogeneous activities whilst concurrently optimizing QoS and power consumption.
	Disadvantage: The strategy of retaining a high-priority job just on the fog node to complete the procedure can generate congestion as the number of end customers in areas of the network grows.
	Simulation/Implementation: used MATLAB R2017a software platform.
	Dataset: -
	Future work: Examine the remaining energy of the fog node, including the time and energy spent processing a task before allocating it to a node. Consider the available memory—the effect of the runtime algorithm on the execution time of tasks.
Abdel et al. [40]	Main concept: Provide an energy-conscious model basis for dealing with timetable computation (TSFC) to improve QOSs required by users.
	Case study: 12 heterogeneous tasks with different lengths.
	App domain: Fog computing applications.
	Advantage: Least energy consumed, Less carbon dioxide emission rates, Minimum makespan.
	Disadvantage: -
	Simulation/Implementation: implemented using java programming.
	Dataset: Two datasets: (1) A set number of VMs (50), as well as 12 work of varying lengths; (2) A work with a 600-foot fixed length.
	Future work: The fog system’s reliant jobs will also be scheduled using MPA. MPA could potentially be utilized to solve problems such as multifunctional knapsacks and DNA fragment repair.
Ali et al. [41]	Main concept: Trust enhancement for efficient task scheduling in mobile cloud environments.
	Case study: -
	App domain: Mobile cloud computing.
	Advantage: Time, energy, and resources are not wasted.
	Disadvantage: Lack of attention to security and security parameters.
	Simulation/Implementation: implemented the algorithm in MATLAB.
	Dataset: -
	Future work: Calculating trust through communication and scheduling through artificial intelligence methods.
Wang et al. [42]	Main concept: Introducing a new distributed RL approach to optimize physical system real-time dynamics sampling policy.
	Case study: -
	App domain: Wireless channel.
	Advantage: Minimizing the total AoI weight and power consumption of all IoT devices.
	Disadvantage: -
	Simulation/Implementation: -
	Dataset: -
	Future work: -
Azizi et al. [15]	Main concept: Reducing the system’s overall power usage while fulfilling the way to complete.
	Case study: -
	App domain: fog computing systems.
	Advantage: Minimizing energy consumption and timeout in all fog nodes.
	Disadvantage: How to classify IoT tasks for processing on fog nodes or cloud servers has not been studied.
	Simulation/Implementation: simulations are coded in C++.
	Dataset: -
	Future work: Improving proposed algorithms for scheduling IoT-related jobs and evaluating their performance against various real-world datasets.
Li et al. [43]	Main concept: Minimizing AoP degradation by optimizing data evacuation, making a decision.
	Case study: -
	App domain: Wireless channel.
	Advantage: Receiving status updates according to data processing in IoT applications.
	Disadvantage: -
	Simulation/Implementation: -
	Dataset: -
	Future work: Extending the architecture of the essay to far more difficult scenarios involving several IoT devices and edge servers.

**Table 17:** Analysis of articles in terms of evaluation parameters in energy efficiency applications.

Study	Deadline	Cost	Latency	Throughput	Effectiveness	Execution Time	Makespan	Energy Consumption
He et al. [24]	✗	✗	✗	✗	✗	✗	✗	✓
Ghanavati et al. [39]	✗	✗	✗	✗	✗	✓	✗	✗
Abdel et al. [40]	✗	✓	✗	✗	✗	✓	✓	✓
Ali et al. [41]	✗	✗	✗	✓	✗	✓	✗	✓
Wang et al. [42]	✗	✗	✓	✗	✗	✗	✗	✓
Azizi et al. [15]	✗	✗	✗	✗	✗	✓	✓	✓
Li et al. [43]	✗	✗	✗	✗	✗	✓	✗	✗

**Table 18:** Categorizing the state-of-the-art in health monitoring applications.

Study	Attributes
Iqbal et al. [11]	Main concept: Monitor patient health conditions.
	Case study: -
	App domain: Wireless network.
	Advantage: Remote patients with cost-effective and trustworthy services.
	Disadvantage: -
	Simulation/Implementation: Python PyCharm Professional 2020 is used in this experiment. MySQL is used to store persistent data. Locust is employed to simulate subjects.
	Dataset: -
	Future work: To increase the efficacy of IoT-based healthcare services, predictive analytics should be combined with IoT to predict vital signs data.
Tariq et al. [12]	Main concept: Developing a new energy-conscious rescheduling and integrating with a nonlinear programming.
	Case study: 12 real benchmarks.
	App domain: Healthcare.
	Advantage: Reducing delays due to rescheduling without compromising energy efficiency.
	Disadvantage: -
	Simulation/Implementation: Simulation environment in MATLAB.
	Dataset: Set of periodic tasks.
	Future work: Considering Quality of Experience (QoE).
Aburukba et al. [44]	Main concept: To decrease latency, use an exploratory method to schedule IoT requests.
	Case study: -
	App domain: Wired and wireless networks.
	Advantage: Minimize delays in fog–cloud hybrid computing.
	Disadvantage: -
	Simulation/Implementation: Lingo software (Lingo, 2017).
	Dataset: -
	Future work: Extend this model to schedule critical requests and examine several objective functions for maximizing utilization of the resources and minimizing latency.
Stavrinides et al. [13]	Main concept: A mixed fog and cloud-aware strategy can be used for dynamic scheduling.
	Case study: -
	App domain: WiFi or mobile network (4G/LTE).
	Advantage: Schedule computationally heavy jobs with low needs for communication in the cloud and connection hard tasks with limited computational needs in the fog.
	Disadvantage: Significant monetary cost.
	Simulation/Implementation: Simulation program in C++.
	Dataset: -
	Future work: In the cloud, fog gaps are used to plan computing processes with minimum connectivity needs. During the scheduling process, it also considers the costs of transmitting data between the sensors and IoT layer’s equipment to virtual servers in the fog layer.
Arshed et al. [45]	Main concept: Providing resource-aware scheduling for the distribution of input program modules to fog devices.
	Case study: -
	App domain: Three-tier fog–cloud architecture.
	Advantage: Maximum use of resources in the fog layer and lower monetary costs of employing cloud storage with the shortest possible execution time.
	Disadvantage: -
	Simulation/Implementation: Simulated in the iFogSim.
	Dataset: -
	Future work: -

**Table 19:** Analysis of articles in terms of evaluation parameters in health monitoring applications.

Study	Deadline	Cost	Latency	Throughput	Effectiveness	Execution Time	Makespan	Energy Consumption
Iqbal et al. [11]	✗	✗	✓	✓	✗	✓	✗	✗
Tariq et al. [12]	✗	✗	✓	✗	✗	✓	✗	✓
Aburukba et al. [44]	✗	✗	✓	✗	✗	✗	✗	✗
Stavrinides et al. [13]	✗	✓	✗	✗	✓	✗	✗	✗
Arshed et al. [45]	✗	✓	✗	✗	✗	✓	✗	✗

**Table 20:** Categorizing the state-of-the-art in other areas of applications.

Study	Attributes
Al-Qerem et al. [46]	Main concept: A new variation in the optimistic concurrency control protocol is reintroduced.
	Case study: -
	App domain: Cloud server and a fog node.
	Advantage: Reduce loss rates, restart rates, and connection delays.
	Disadvantage: The present work is only related to readable transactions and updates.
	Simulation/Implementation: Use of the iFogSim simulation tool.
	Dataset: -
	Future work: The ineffectiveness of the validation process. This is performed on the server using standard protocols. Many unnecessary transaction restarts and aborts will occur in the cloud.
Pendit et al. [16]	Main concept: Make a task-scheduling scheme that is based on RL.
	Case study: -
	App domain: Neural networks.
	Advantage: Efficient resource usage and task execution time minimization, as well as a significant reduction in communication expenses.
	Disadvantage: -
	Simulation/Implementation: Experiment with different lengths of instructions (1000–10,000).
	Dataset: -
	Future work: It is necessary to investigate the impact of various RL algorithms for scheduling tasks in the IoT context, such as prioritized deep Q-network (DQN), double DQN, and others.
Potu et al. [47]	Main concept: In fog computing, an Extended Particle Swarm Optimization (EPSO) method was developed to enhance resource efficiency and reduce the time spent completing tasks.
	Case study: Fog computing
	App domain: Cloud–fog environment
	Advantage: Improve resource efficiency and minimize time spent completing tasks.
	Disadvantage: While doing IoT of work in fog nodes helps minimize latency, it can also lead to greater fog node energy usage.
	Simulation/Implementation: iFogSim and Eclipse editor were used to implement Java.
	Dataset: From the 200 to 500 tasks, l7 datasets were created.
	Future work: Design a strategy for exchanging latency and energy consumption using parallel metaheuristic algorithms.
Kandan et al. [48]	Main concept: Designing a new optimization-based job scheduling technique for resource management and allocation in a cloud computing environment.
	Case study: -
	App domain: IoT cloud environment.
	Advantage: Minimum flow time, minimal lateness.
	Disadvantage: -
	Simulation/Implementation: Simulates Aquila’s behaviors.
	Dataset: -
	Future work: To properly schedule jobs in the IoT cloud, a combination of these two metaheuristic algorithms and deep learning algorithms might be offered.
Stavrinides et al. [18]	Main concept: Introduce a dynamic scheduling algorithm using partial calculations to use scheduling gaps to achieve real-time scheduling.
	Case study: -
	App domain: Fog computing environment.
	Advantage: Take into account the consequences of global error propagation between process component activities.
	Disadvantage: Overhead not checked.
	Simulation/Implementation: Simulation program in C++.
	Dataset: -
	Future work: The proposed scheduling technique is implemented in a three-tier environment. In circumstances of high workload, more cloud resources are used to schedule IoT workflows.
Sheng et al. [49]	Main concept: Provide a policy-based REINFORCE algorithm for scheduling tasks based on tasks and heterogeneity of resources in the EC.
	Case study: -
	App domain: Edge computing.
	Advantage: Optimize the order of work execution and job allocation jointly.
	Disadvantage: -
	Simulation/Implementation: Simulation in Python 3.
	Dataset: -
	Future work: Common cloud computing and edge computing taking into account communication latency.
Attiya et al. [50]	Main concept: To discover the best strategy for scheduling IoT applications, the ChOA was combined with the MPA and the Disruptor Operator.
	Case study: There are 1000 tasks in the simulated workload, ranging in length from 1000 to 20,000 MI. The actual workload is made up of 1000 jobs ranging in duration from 1000 to 20,000 MI.
	App domain: Fog computing.
	Advantage: Increasing fog processing power and optimizing the scheduling of IoT application tasks.
	Disadvantage: -
	Simulation/Implementation: Implemented using CloudSim
	Dataset: The “Parallel workload Archives” contains authentic datasets created by NASA Ames iPCS/860 and HPC2N.
	Future work: In a real fog computing system, implement the CHMPAD scheduling algorithm. It can also be used to handle more difficult optimization problems, adjust quadratic characteristics, vehicle routing, assembly line tuning, and healthcare planning, as well as to make other enhancements and advancements.

**Table 21:** Analysis of articles in terms of evaluation parameters in other application areas.

Research	Deadline	Cost	Latency	Throughput	Effectiveness	Execution Time	Makespan	Energy Consumption
Al-Qerem et al. [46]	✗	✗	✓	✗	✓	✗	✗	✗
Pendit et al. [16]	✗	✓	✓	✗	✗	✓	✗	✗
Potu et al. [47]	✗	✓	✗	✗	✗	✗	✓	✗
Kandan et al. [48]	✗	✗	✓	✓	✗	✓	✓	✗
Stavrinides et al. [18]	✗	✗	✗	✗	✗	✓	✓	✗
Sheng et al. [49]	✗	✗	✗	✗	✓	✗	✗	✗
Attiya et al. [50]	✗	✗	✗	✓	✗	✗	✓	✗

## Data Availability

Not applicable.
